# Stem Cell’s Secretome Delivery Systems

**DOI:** 10.34172/apb.2023.027

**Published:** 2022-01-03

**Authors:** Abd. Kakhar Umar

**Affiliations:** ^1^Department of Pharmaceutics and Industrial Pharmacy, Faculty of Pharmaceutical Sciences, Chulalongkorn University, Bangkok 10330, Thailand.; ^2^Department of Pharmaceutics and Pharmaceutical Technology, Faculty of Pharmacy, Universitas Padjadjaran, Jatinangor 45363, Indonesia.

**Keywords:** Stem cell's secretome, Secretome delivery systems, Cell-free therapy

## Abstract

Stem cells’ secretome contains biomolecules that are ready to give therapeutic activities. However, the biomolecules should not be administered directly because of their in vivo instability. They can be degraded by enzymes or seep into other tissues. There have been some advancements in localized and stabilized secretome delivery systems, which have increased their effectiveness. Fibrous, in situ, or viscoelastic hydrogel, sponge-scaffold, bead powder/ suspension, and bio-mimetic coating can maintain secretome retention in the target tissue and prolong the therapy by sustained release. Porosity, young’s modulus, surface charge, interfacial interaction, particle size, adhesiveness, water absorption ability, in situ gel/film, and viscoelasticity of the preparation significantly affect the quality, quantity, and efficacy of the secretome. Therefore, the dosage forms, base materials, and characteristics of each system need to be examined to develop a more optimal secretome delivery system. This article discusses the clinical obstacles and potential solutions for secretome delivery, characterization of delivery systems, and devices used or potentially used in secretome delivery for therapeutic applications. This article concludes that secretome delivery for various organ therapies necessitates the use of different delivery systems and bases. Coating, muco-, and cell-adhesive systems are required for systemic delivery and to prevent metabolism. The lyophilized form is required for inhalational delivery, and the lipophilic system can deliver secretomes across the blood-brain barrier. Nano-sized encapsulation and surface-modified systems can deliver secretome to the liver and kidney. These dosage forms can be administered using devices such as a sprayer, eye drop, inhaler, syringe, and implant to improve their efficacy through dosing, direct delivery to target tissues, preserving stability and sterility, and reducing the immune response.

## Introduction

 Treatment with stem cells is becoming increasingly common for regenerative therapy, implantation, and protein supply.^1-6^ However, the application of stem cells still has many drawbacks. Stem cells cannot be grown in large numbers or for an extended period of time.^7,8^ The small amount in the culture medium makes it difficult to isolate and purify.^9,10^ Immune system resistance, tumor or cancer growth, atherogenesis, and arrhythmogenesis are possible outcomes.^11-15^ Some recent evidence also suggests that the therapeutic effect does not result from transdifferentiation and engraftment of stem cells but by releasing paracrine factors such as cytokines, growth factors, and exosomes. This biomolecule is called the secretome and acts as a communication system between cells. Therapy using secretome is said to be better than cell-based therapy.^16-22^ As a result, there has been growing interest in the use of secretome in the clinical field, primarily because it has a few advantages over the conventional use of stem cells in regenerative pharmaceutical treatment, including ease of delivery, decreased concerns for oncogenic potential associated with stem cell use, and the absence of immunogenic response enabling allogeneic or off-the-shelf therapy.^5,19,21,23^

 Direct administration of the secretome may decrease its efficacy. Since the secretome depletes rapidly due to enzymatic degradation or migrates to other tissues, it is often given in large amounts or repeated doses.^24^ Administration in large doses can cause dose-dependent cytotoxicity.^25^ Secretome can quickly spread to other tissues/organs such as the liver, lung, spleen, kidney, heart, muscle, and possibly brain within 30 minutes of injection.^26,27^ As a result, a controlled and localized delivery system is needed to improve the secretome’s retention and efficacy in the target tissue. However, the secretome’s modulation effect is significantly influenced by the stiffness and nature of the delivery system, so the selection of the base needs to be considered carefully.^28^ Using an appropriate base can increase therapy’s effectiveness through a synergistic mechanism of action with the secretome component.^28-30^ In vivo stability and delivery of the secretome to the target tissue or through the blood-brain barrier to the central nervous system is possibly achieved with a suitable delivery system.^31^

 Several dosage formulations, such as fibrous, in situ, or viscoelastic hydrogel, beads powder/suspension, cell-mimicking coatings, and sponge-scaffold, have been investigated and proven to be successful confidential provisions. Some of them can even selectively regulate the release of proteins in the secretome. Porosity, young’s modulus, surface charge, interfacial interaction, particle size, adhesiveness, water absorption ability, in situ gel/film, and viscoelasticity of the preparation significantly affect the quality, quantity, and efficacy of the secretome.^31-39^ Therefore, this review discusses the clinical obstacles and potential solutions for secretome delivery, dosage forms, compositions, and characteristics of the secretome delivery systems for therapeutic applications and devices used or potentially used to improve the effectiveness of secretome therapy.

###  Secretome and its in vivo stability

**Figure 1:**
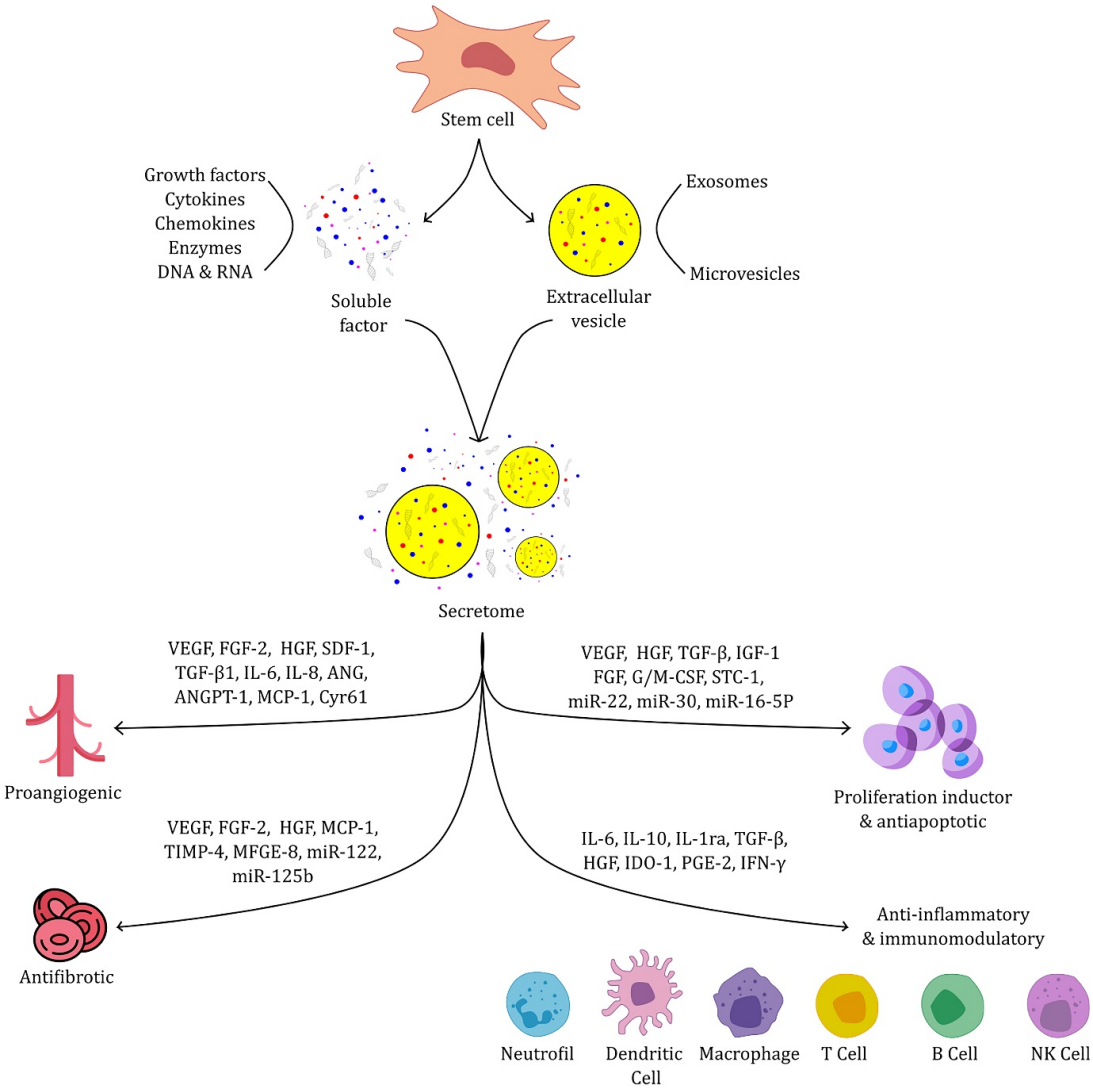
Illustration of the origin of secretome and its therapeutic activity.

 Secretome is a collection of various bioactive factors that work synergistically to induce therapeutic effects.^27,40-42^ Secretome contains growth factors, cell adhesion molecules, cytokines, microvesicles, chemokines, exosomes, hormones, serum and extracellular matrices proteins, proteases, and lipid mediators.^22,42^ Illustration of the origin of the secretome and its therapeutic activity can be seen in Figure 1. Various studies have been carried out to optimize the levels of specific proteins desired by adjusting the stem cell medium’s culture conditions. The conditioned medium can be used to supply protein/factors to provide the desired therapeutic effect. However, administering the secretome directly to the systemic or local tissues will cause rapid clearance or seep into other tissues and eventually accumulate in the kidney, spleen, or liver, requiring repeated doses to prolong therapy.^43,44^ For this reason, a delivery system is required to extend the retention of the secretome in the therapeutic target tissue and regulate its release, thereby reducing repeated dosing.

###  Clinical obstacles and solution for stem cell’s secretome delivery

**Figure 2:**
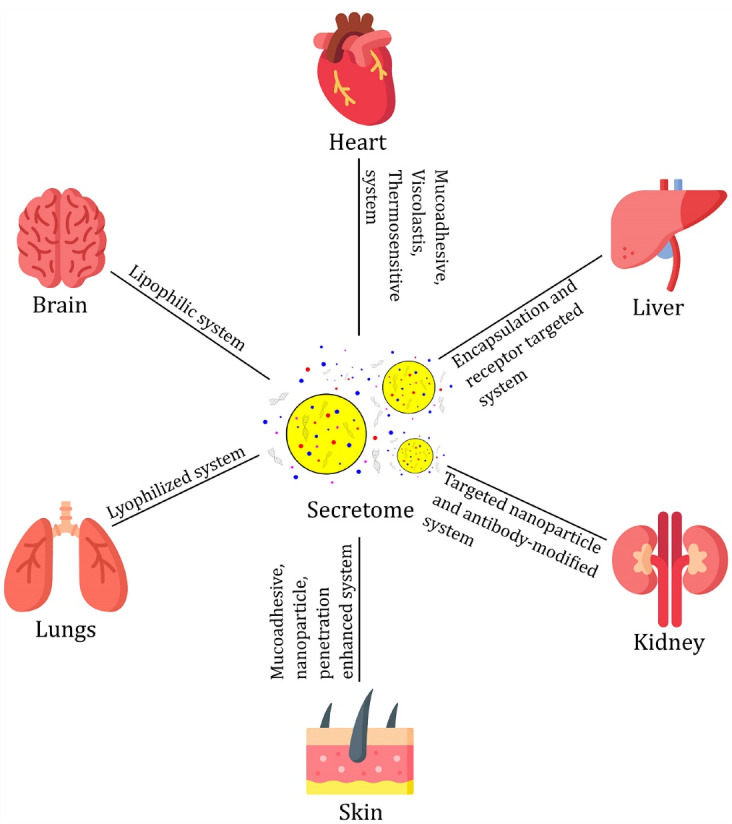
Potential solutions for organ-targeted secretome delivery.

 It is well known that stem cells exert a therapeutic effect through the help of paracrine factors. These soluble biomolecules can be used to develop “cell-free” regenerative medicine. Secretome has been widely used in various clinical pathological conditions, including disorders of the brain, lungs, heart, liver, kidneys, skin, etc.^45–49^ However, in its application, there are still some shortcomings that lead to a decrease in secretome effectiveness. Several attempts have been made based on the target organ of therapy (see Figure 2).

 In the treatment of brain disease, secretome has outstanding potential, especially in balancing the levels or supplying certain factors in the nervous system for regenerative therapy.^50,51^ However, the application of secretome in neuroregenerative therapy has obstacles because the damaged tissue is difficult to target through systemic administration, and direct infusion of secretome can reduce its efficacy due to the narrow therapeutic window.^52^ Due to its polar nature, the secretome is difficult to penetrate the blood-brain barrier. For this reason, the secretome must be applied directly to the brain or administered indirectly using a delivery system that can diffuse to the brain.^51-53^ The lipophilic system can deliver the secretome across the blood-brain barrier.

 In the systemic circulation, the contents of the secretome can be metabolized, especially those targeted for hepatic therapy. The liver performs metabolic, immunological, and endocrine functions that are very important for the body. Blood circulation in the liver occurs through a network of permeable discontinuous capillaries known as sinusoids, where the small blood vessels (5–10 μm) have rows of hepatocytes radiating between them. Within the sinusoidal capillaries, there are Kupffer cells that are responsible for phagocytic activity in the liver. Therefore, secretome delivery to the liver requires protection through a targeted encapsulation system. This method is better than affecting the work of enzymes in reducing metabolic activity because it can cause side effects. In addition, the encapsulation system must be nano-sized so that it can pass through capillary blood vessels and be efficiently absorbed by liver cells.^54^

 Secretomes that are in the systemic circulation system will quickly spread to organs and tissues.^24^ Therefore, therapy that requires the secretome to arrive and be present in the desired amount in a particular organ requires a targeted delivery system with muco- and cell-adhesive properties. This delivery system is required for organ-targeted therapies such as those for the liver and kidneys.^29,55^

 Secretome contains proangiogenic, antiapoptotic, antifibrotic, and anti-inflammatory and immunomodulatory properties. However, an excess of one or more unwanted or needed factors can cause side effects.^22^ To keep levels of adverse factors low, the culture medium of the stem cells must be controlled, or the secretome delivery system must be able to filter the release of the components they contain. This selective release system certainly requires a selectively permeable membrane layer.^35^ Therefore, the selection of basic components needs to be considered.

###  Delivery systems for secretome

 The secretome delivery system can be either its conditioned medium^52^ or a separately developed system.^25^ Here are some delivery systems for secretome (see Table 1).

**Table 1:** Delivery systems and base components used in increasing the effectiveness of secretome.

**Secretome Source**	**Delivery System**	**Base Component**	**Objective**	**Ref**
Mesenchymal stem cells	Injectable hydrogel	Type I bovine collagen and low-molecular-weight hyaluronic acid or collagen and polyethylene glycol.	Controlled delivery system	^52^
Adipose-derived stem cells	Hydrogel	Synthetic PIC	Tunable matrix for controlling secretome release	^34^
Chondrocytes Derived stem cells	Biodegradable porous sponge cartilage scaffold	Sponge cartilage bovine scaffold	Promote cartilage survival or differentiation and inducing growth factor	^38^
Mesenchymal stem cells	Nanocomposite-hydrogel	Poly-L-lactide nanoparticles, gelatin, and hyaluronic acid	Controlled delivery system	^25^
Adipose-derived stem cells	Peptide nanofiber hydrogel	E_2_(SL)_6_E_2_GRGDS peptide	Controlled delivery system	^56^
Mesenchymal stem cells	Sponge	Alginate	Controlled delivery system	^39^
Adipose-derived stem cells	Injectable hydrogel	PNIPAM, polyethylene glycol, and peptide	Controlled delivery system and proangiogenic secretion	^57^
Bone marrow-derived stem cells	Electrospun fibers	Gelatin and polycaprolactone	Controlled delivery system	^37^
Endothelial progenitor cells	Shear-thinning hydrogels	Adamantane-modified hyaluronic acid and β-cyclodextrin-modified hyaluronic acid	Controlled delivery system	^58,59^
Mesenchymal stem cells	Viscoelastic gel	Hyaluronic acid and chondroitin sulfate	A synergistic delivery system for corneal wound healing	^30^
Marrow isolated adult multilineage-inducible cells	Pharmacologically active microcarriers hydrogel	PLGA, poloxamer (P188), and Si-HPMC	Protect and deliver the cargo through the blood-brain barrier sustainedly	^31^
Mesenchymal stem cells	Secretome crosslinked hydrogel	Methacrylate hyaluronic acid	Intrauterine drug delivery system with sustained release	^60^
Human cardiac stem cells	Theracyte devices	-	Implantable cell and sustained release of secretome	^61^
Adipose-derived stem cells	TIPS microcarriers	PLGA	Targeted delivery system	^36^
Human adipose-derived stem cells	Injectable physically crosslinked nanocomposite hydrogels	Laponite XLG and gelatin type A	Biocompatible localized delivery system	^29^
hBMSC	Biomimetic mineralized collagen scaffolds	Fibrillated and mineralized collagen type 1 and 1-ethyl-3-(3-dimethyl aminopropyl) carbodiimide	Sustained release and angiogenic delivery base for bone grafting	^62^
Bone marrow stem cells	Semipermeable cellulose beads	Cellulose sulfate	Selective and continuous release	^35^
Bone marrow stem cells	Macroporous scaffolds	PLG	Controlling cell phenotype	^63^
Human umbilical vein endothelial cells	Nanoparticle suspension	α-methoxy-ω-2-(N,N-diethanolamine)ethyl-poly(ethylene glycol), polylactide, and PMDA	Controlled release delivery system	^64^
Cardiac stem cells	CMMP	Poly(lactic-co-glycolic acid) and polyvinyl alcohol	Controlled release delivery system which not induce an immune response	^33^
Mesenchymal stem cells	Photocrosslinkable hydrogel	Alginate and RGD peptide	Targeted and Prolonged release delivery system	^65^
Human umbilical vein endothelial cells	Photocrosslinkable hydrogel	Gelatin methacryloyl	Controlled release delivery system	^66^
Bone marrow stromal cell	Scaffold	Poly(lactic-co-glycolic acid) and poly(ethylene glycol)	Controlled release delivery system	^67^

TIPS, Thermally-induced phase separation; PIC, polyisocyanide; PNIPAM, poly(N-isopropyl acrylamide); PLGA, Poly(lactic-co-glycolic acid); Si-HPMC, silanized-hydroxypropyl methylcellulose; hBMSC, Human bone marrow stromal cells; PLG, poly(lactide-co-glycolide); PMDA, pyromellitic dianhydride; CMMP, Cell-mimicking microparticle; RGD, arginine-glycine-aspartic acid.

 Based on Table 1, it can be seen that the secretome delivery system is generally in the form of polymeric gel and sponge-scaffold. It depends on the therapeutic purpose. A polymeric gel is often used to guide stem cells to generate more complex proteins. Furthermore, the polymeric gel can stimulate proliferation and inhibit stem cell aging, resulting in increased secretome production. Meanwhile, the sponge-scaffold method is commonly used in wound care, especially for bone injury, since it facilitates tissue adhesion. Several other unique delivery systems have been used for secretome delivery.

###  Cell-mimicking coating

 Recently, it has been known that stem cells do not have a therapeutic effect through tissue division and replacement but rather from the factors they secrete.^68-70^ However, further studies have shown that cell-to-cell contact between donor cells and host cells plays an important role.^71^ The use of live stem cells for transplantation has significant drawbacks. Live stem cells should be carefully cryo-preserved and thawed before administration. Each treatment given will also significantly affect the outcome of therapy. Besides, live stem cells can also induce tumor formation and immunity. For this reason, the use of delivery that can be similar to the original cell will be beneficial.^33^

 Poly(lactic-co-glycolic acid) (PLGA) has been widely used as a protein carrier and skeleton for cell-mimicking coatings.^72-75^ This cell-mimicking microparticle (CMMP) has been successfully used as a secretome delivery system without inducing an immune response. The particle size is similar to the original cardiac stem cell, ± 20 µm. The double coating (PLGA and cell membrane itself) does not interfere with releasing the secretome it carries. Freezing below -80°C and thawing in water did not affect the surface’s coating membrane, size, or antigen expression. The CMMP also shows rolling and traveling behavior on cardiomyocytes to which it attaches, suggesting the biointerfacing between them. CMMP was also degraded, with only a small amount remaining in the hearts of mice 28 days after administration. The secretome can improve pump function, angiogenesis, and viable myocardium while decreasing apoptosis and wound size by CMMP delivery.^33^

###  Polymeric gel

#### Alginate

 Alginate is biocompatible, biodegradable, and tunable, making it easy to deliver biomolecules.^76,77^ Alginate dressings can collect wound moisture in the dry state to form gels, provide a dry wound with a physiologically moist atmosphere and decrease bacterial infections, facilitating accelerated re-epithelialization and granulation tissue development.^78^ Photo crosslinked alginate hydrogel with arginine-glycine-aspartic acid (RGD) peptides (RGD hydrogel) linked to the backbone was reported to successfully encapsulate the bound extracellular vesicle (EV) in a conditioned medium. The particle size of the EV encapsulated in the non-RGD alginate hydrogel tends to increase due to the formation of EV aggregates. The use of 4% RGD hydrogel was the best in regulating EV release and integrity. Release studies show that RGD hydrogel releases EV after 3-5 days of application. The volume of regenerated bone appears to be more significant between weeks 4 and 8 and suggests effective retention and EV distribution. After eight weeks, significant changes in bone repair were seen in the group receiving the hydrogel RGD compared to the alginate hydrogel or RGD group alone.^65^

#### Collagen

 Collagen hydrogels can improve cell adhesion, survival, and proliferation, allowing translational stem cell medicine to reliable donor-derived stem cell isolation, growth, and banking.^79-81^ Chierchia et al discovered that a secretome isolated from conditioned media (collagen hydrogel) would counteract oxidative stress caused by the dopaminergic-selective toxin 6-OHDA. The secretome in collagen hydrogel can be active for 48 hours and reduce nerve cell death, with optimal protective effect when not diluted. The mixture of collagen and polyethylene glycol 2000 (PEG2000) showed a higher percentage of cell recovery than the mixture of collagen and hyaluronic acid (HA), although it was not significant. The optimal collagen concentration is 1.2-1.8 mg/mL, PEG2000 of 0.6 mg/mL, and HA of 2.5 mg/mL.^52^ Research conducted by Joshi et al shows that using collagen from mice is significantly better in increasing the proliferation of human bone marrow mesenchymal stem cells than using collagen from humans. Of course, this will affect the quality and quantity of secretome content generated.^82^

#### E_2_(SL)_6_E_2_ GRGDS peptide

**Figure 3:**
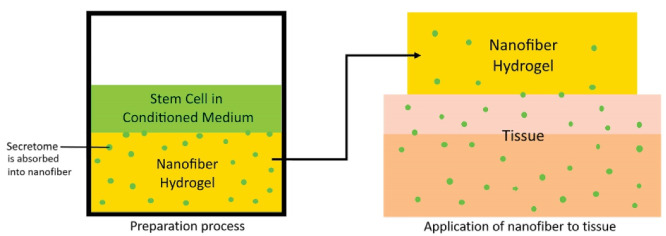
Preparation of embedded secretome in nanofiber peptide hydrogel and release mechanism of secretome from nanofiber peptide hydrogel to target tissue.

 Peptide hydrogels show good potential as therapeutic materials, including cell scaffolds and protein delivery systems. These peptides form a nanofiber matrix hydrogel upon exposure to ions, such as Mg^2+^. This peptide hydrogel can act like a sponge where the secretome excreted by the stem cells into the culture medium can diffuse through the hydrogel’s permeable membrane. Furthermore, hydrogel peptides can be used as a therapy in a cell-free manner.^56^ Illustration of secretome preparation in peptide hydrogel can be seen in Figure 3.

 Nanofiber peptide hydrogel (NPH) has viscoelastic properties to be delivered by injection into the target tissue. The NPH becomes stiff after the pressure is removed and remains on the target tissue for 24 hours. Since BPH does not disappear instantly, the rigidity of NPH means that secretome release occurs slowly.^56^

#### Gelatin

 Gelatin is beneficial in delivering biomolecules because it contains sequences of RGD, which is favorable for cell attachment and proliferation.^66^ Combining gelatin and Laponite can be used as a localized delivery system for the secretome (cell-free). The stability and local retention of the system was better at higher Laponite concentrations. The optimal concentration is 2% Laponite and 5% gelatin. These preparations are biocompatible in vitro and in vivo. This injectable hydrogel is also reported to have a synergistic therapeutic effect with the secretome in improving heart function (acute myocardial infarction) through angiogenesis. It is better than administering the secretome solution itself.^29^ Gelatin methacryloyl (GelMA) hydrogel has also successfully regulated the sustained release of exosomes. It helps accelerate wound healing by promoting re-epithelialization, collagen deposition, and angiogenesis. The animal group that received GelMA-exosomes showed a faster wound closure than the GelMA-exosomes and control group.^66^

#### Hyaluronic acid

 HA is a natural material that can form complexes with other polymers and produce more favorable physical characteristics. Adamantane-cyclodextrin crosslinked HA has shear-thinning and viscoelastic properties. This system has also been shown to increase retention and localize endothelial progenitor cells. The shear-thinning gel (STG) system can also regulate the release of EVs for up to 21 days. EVs loaded into STG showed 82% better hemodynamic function improvement than control (PBS) and EVs alone. The system was successful in decreasing end-diastolic and systolic but overall increased stroke volume. The STG system itself also shows increased recruitment of inflammatory cells.^58,59^

 Besides its application as a delivery system, hyaluronic acid has also been shown to heal corneal wounds synergistically. The secretome of MSCs delivered in the HA-chondroitin sulfate system demonstrates reduced scar formation, neovascularization, and bleeding following alkaline corneal burns.^30^ Crosslinked HA-secretome also shows excellent characteristics as an intrauterine controlled-release delivery system. The system is more stable and biodegradable. The system succeeds in releasing the secretome slowly and can last up to two estrous cycles. Besides, crosslinked HA-secretome gel is a more effective infertility treatment than the gel and secretome alone.^60^

#### Poly-L-lactide acid

 Poly-L-lactide Acid (PLA) is a biodegradable thermoplastic polymer that has been widely used in nanoparticle delivery systems. PLA is degraded by hydrolysis to produce lactic acid, which is easily eliminated by the body.^83^ Shoma Suresh et al discovered that PLA would overlay protein from the secretome to form nanoparticles ranging in size from 407.95 nm to 618.55 nm with a robust dispersion mechanism. The zeta potential ranges from -30.75 mV to -34.35 mV. Protein release can take up to 8 days. Implantation of PLA nanoparticles in gelatin-hyaluronic acid hydrogel (NP-H) prevents burst release of protein and prolongs protein release by up to 9 days. The different culture mediums used also showed different cell proliferation-inducing abilities. Serum-containing conditioned medium in NP-H induces metabolite activity of fibroblast cells up to 98.2% for 24 hours and is significant against other serums used as a culture medium.^25^

#### Polyisocyanide

 Synthetic polymers can be modified to produce the desired characteristics so that they are superior to natural polymers. Polyisocyanide (PIC) has been reported to have an in situ gel property. It can form a gel at temperatures above 15°C and control the secretome’s release. The length of the polymer chain determines this property.^34^ PIC is also reported to modulate cell adhesion capacity, cell motility, proliferation, vascularization, and cytocompatibility.^84,85^ The type and content of the secretome produced during the culture can be tuned by this polymer.^34^

 A PIC with a reduced chain length forms a softer gel. The addition of Gly-Arg-Gly-Asp-Ser (RGD) peptide also reduced the stiffness of the gel. In a conditioned medium containing PIC, the levels of eotaxin, GRO, IL-6, IL-7, IL-8, IL-10, MCP-1, and vascular endothelial growth factor (VEGF) increased. These proteins are produced more on the RGD-PIC gel. The levels of IL-10 are very high in the conditioned medium containing the RGD-PIC and show a significant role in fibroblast cell proliferation. A fast wound closure (close to 100%) occurs 48 hours after the RGD-PIC-secretome is administered (without dissolved).^34^

#### Poly(N-isopropylacrylamide)

 Like other synthetic polymers, poly (n-isopropyl acrylamide) (PNIPAM) can be modified by adding peptide chains to produce a cell adhesive and thermoresponsive microenvironment as well as a custom protein generator.^86-88^ The peptide chain on PNIPAM acts as a cell-adhesive spacer. The system managed to attach and maintain the preparation on the myocardium for 16 days with significant retention compared to the control group. Increased levels of PNIPAM correlate with the thickness and stiffness of the gel. Cell proliferation is more optimal in the gel containing 1.25 %wt PNIPAM (SHIELD-1.25). Levels of growth factor are also relatively higher on the SHIELD-1.25 gel.^57^

#### Poly(lactic-co-glycolic acid)

 PLGA is known to have a proangiogenic effect through its metabolites (lactic and glycolate), which can support wound healing and promote endothelial cell migration in vitro.^89,90^ According to studies, PLGA has also effectively distributed certain biomolecules,^91-94^ including secretomes, through the blood-brain barrier and then into the central nervous system.^31^ Kandalam et al succeeded in developing a delivery system of brain-derived neurotrophic factor (BDNF) and secretome as a neural cell differentiation therapy. They complexed PLGA and poloxamer 188 (PAM) to form spherical particles with an average diameter of 33 ± 12 µm. With a fibronectin layer on its surface, the PAM system has a zeta potential of 41 ± 6,6 mV with an entrapment efficiency of 76,9 ± 3,3%. In vitro release studies showed that BDNF was released slowly in a linear pattern without burst release for 40 days. The PAM system can also maintain cell viability for up to 7 days in vitro.^31^

#### Cellulose derivates

 PAM-stem cell aggregation will last longer with the aid of silanization-hydroxypropyl methylcellulose (Si-HPMC) 2% as the outer matrix (hydrogel base), which correlates with cell viability. Some growth factor expression also increased significantly. Besides, Si-HPMC is biodegradable and forms an in situ gel at physiological pH, so it is best used as an outermost matrix in localized drug delivery systems.^31^ Cellulose sulfate has also been developed as a biocompatible microenvironment for cells. This system can maintain cell viability and extend the secretion of the therapeutic molecule.^95,96^ Many biomolecules, such as insulin, cytokines, antibodies, and enzymes, have been identified as having an outflux.^97,98^

###  Micro-nanoparticle powder/suspension

#### Cellulose sulfate beads

 Cellulose sulfate beads can be used as a coating for live stem cells and regulate the release of their secretions. Each bead can cover 800 to 1000 cells with an average bead size of 750 µm ± 25 µm. After the coating process, it was seen that only 65% of the cells were still alive, but the ones that were still alive survived over time. The EVs excreted by these beads are smaller and uniform in size, with seven times more in levels than the EV from the 2D medium with an EV size of 123.9 ± 21.8 nm. The porous size of the beads that behave as filters may impact this.^35^

#### Poly(lactic-co-glycolic acid) microcarrier

 Simitzi et al succeeded in making porous biodegradable microcarriers from PLGA using the thermally induced phase separation (TIPS) method. TIPS is a simple, robust method that does not require the addition of porogen materials. The types of PLGA used were Purasorb PDLG7507, PDLG5010, and PDLG8531, with a lactide: glycolide ratio of 75:25, 50:50, and 85:15, respectively. The three types of PLGA form microcarriers with an average size of 300 µm, where PDLG7507 forms the largest pore size. These pores can help the mobility of cells and their secretions. This type of PLGA also supports better cell adhesion and forms a single layer on each microcarriers’ surface.^36^

 The particle size and surface properties influence the internalization of the PLGA microcarrier. MSCs are said to internalize particles with positively charged surfaces more efficiently than those with negatively charged surfaces. The addition of a carboxylic acid group to the end chain of PLGA produces a negatively charged surface. The surface charge can be modified by absorbing polycationic polymers (poly-L-lysine) or conjugating antibodies or lipids (N-hydroxysuccinimide-biotin). With this modification, MSCs can internalize PLGA as small as 1 μm. This system can be used for tracking stem cells and controlling secretome production.^99^

#### α-Methoxy-ω-2-(N,N-diethanolamino)ethyl-poly(ethylene glycol) – polylactide – pyromellitic dianhydride (mE2N-PLA_2_-PMDA_2_)

 The particle size of the synthetic polymer mE2N-PLA2-PMDA2 nanoparticles (NPs) is 120 ± 20 nm at 58.5% of its total volume with a zeta potential of -20.30 ± 2.61 mV. The encapsulation efficiency reaches more than 64% of the whole secretome cytokine. The release profile of each type of protein in the secretome is different. VEGF had a cumulative release of 70%, MCP-1 of 26%, and was very slow in IL8 and PDGF-BB observed on day 14. Blood perfusion was significantly higher in the group that received the nanoparticle-coated conditioned medium (CM-NPs) than the CM alone and the control group in vivo. At week 2, replacement of necrotic tissue and formation of new vascular systems was found in the CM and CM-NPs groups. According to the findings, the mE2N-PLA2-PMDA2 nanocarrier effectively facilitates ischemic tissue regeneration compared to direct secretome administration.^64^

###  Sponge-scaffold

 The scaffold system can be used as a growth medium as well as a good dosage delivery system. This system can even play a direct role in tissue adhesion, such as treating bone defects. Besides, the administration of secretome by transplant showed better improvement than direct administration of secretome one to three times a day.^100^ Another benefit is that the scaffold can be shaped to make it more comfortable and suitable for application to the tissue.^37^

#### Collagen scaffolds

 On the one hand, biomaterial scaffolds have the inherent capacity to attract cells with regenerative potential in situ. They also provide an adequate environment for proliferation and differentiation, allowing neo-tissue growth and remodeling without extracorporeal cell seeding and cultivation. Because of their interconnected-porous microarchitecture, mineralized collagen scaffolds provide a suitable condition for cell regeneration. They can reach ≈90% porosity and a pore size of 180 ± 12 μm.^101^ This nanocomposite was created using a biomimetic method of synchronous collagen fibril reassembly and mineralization. Nanocrystalline hydroxyapatite (65–67 wt%) is strongly linked to collagen type I matrix (28–30 wt%), which perfectly matches the natural extracellular bone matrix. After freeze-drying, scaffolds are chemically crosslinked with EDC (1-ethyl-3-(3-dimethyl aminopropyl) carbodiimide), leading to highly elastic scaffolds in a wet state with a compressive modulus ≈28 kPa at 50% uniaxial compression. EDC is used to chemically crosslink the scaffolds, resulting in highly elastic scaffolds in the fluid condition with a compressive modulus of 28 kPa at 50% uniaxial compression.^62,101^

 Mineralized collagen scaffold (diameter: 6 mm, height: 8 mm), in the presence of hypoxia conditioned medium (HCM), successfully fascinated human bone marrow stromal cells (hBMSC) into the scaffold after three days by surface inoculation. The migration of hBMSC is affected by HCM, and its increase correlates with the concentration of HCM. Human serum as an HCM solvent also induces more and deeper hBMSC migration up to 2.0 mm than alginate-based depot (ABD). VEGF release was also significantly inhibited by the use of ABD compared to human serum. The formation of well-developed tubular networks showed a strong angiogenic potential of all scaffolds. Interestingly, the prevascular structure appeared more prominent on the ABD-modified scaffold.^62^ It is also claimed that the collagen scaffold’s natural properties should be studied further because it will be difficult to adjust the shape and size to fit when applied to a bone, particularly larger defects.^102^

#### Electrospun fibers scaffold

 The MSC secretome’s delivery via the electrospun fibers scaffold (EFS) improves the corneal epithelial and stromal tissue in severe wounds while avoiding scarring and neovascularization. The hEGF is produced up to 5 times more in this system than in a 2D culture environment. High hEGF levels cause the induction of proliferation in fibroblast cells to occur more quickly, which correlates with the wound’s rapid closure. Carter et al discovered that an EFS system made of polycaprolactone and gelatine in a 1: 1 ratio has mechanical properties that match Young’s modulus of native corneal tissue, allowing it to support the growth of MSC and secretome secretions. The EFS system also releases the secretome sustainably and promotes more effective healing of corneal wounds.^37^

#### Poly(lactide-co-glycolide) scaffold

 Poly (lactide-co-glycolide) (PLG) macroporous scaffolds can be made via gas foaming/particulate leaching. The decellularized matrix (DM) of bone marrow-derived stem cells can be incorporated onto this macroporous scaffold and aids in the expression of osteocalcin and bone sialoprotein from MSCs, both of which are markers of mature osteoblast activity. The DM protein is uniformly distributed on the scaffold surface. The DM-coated scaffold has a rougher surface than the uncoated scaffold. The DM-coated scaffold’s porosity was also reduced compared to the uncoated scaffold, but the difference was insignificant. Total DNA quantification revealed that cell viability was higher in the DM-coated scaffold. Calcium deposition and ALP expression were also increased in the DM-coated scaffold implanted with osteogenically induced MSCs. The system also produced higher vessel density after two weeks of in vivo testing on mice.^63^

#### Sponge cartilage bovine scaffold

 Cartilage is avascular, anisotropic, and aneural tissue, making it difficult to regenerate.^103^ The sponge-scaffold technique has been used extensively in the treatment of cartilage damage. The method of producing bovine scaffold sponge cartilage is simpler and cheaper. These sponge cartilage bovine scaffolds can be made from cartilage of the femoral head and condyles of certified healthy Ongole Cattle aged 24 months.^38^ The sponge-scaffold pores had a diameter range between 50 µm–150 µm. The pore size of 100 - 300 µm provides a conducive cell adhesion and proliferation environment and is suitable for articular cartilage engineering.^104,105^ If the porous diameter is too small, it will inhibit the mobility of the signaling factor, thereby affecting cell viability.^106^ Decellularized sponge-scaffold had a lower percentage of pores (88.93 ± 4.18%) than the sponge-scaffold with cells (90.07 ± 4.64%), but the difference is not significant (p = 0.473). The proliferation of chondrocytes is better in decellularized sponge-scaffold.^107^ The addition of a secretome to the scaffold improved regeneration from hyaline-like cartilage significantly.^38^

#### Sponge-like alginate

 According to Bari et al, the sponge-like alginate system can control protein and lipid secretomes’ release for up to 48 hours. Wounds treated with this system did not show any complications or infection. This phenomenon may be due to the sponge-like alginate’s ability to absorb excess wound moisture rather than direct administration of a secretome, which indicates prolonged inflammation. Treatment with this system is also faster than direct secretome administration. On day 14, the wound treated with this system showed marked vascularization, collagen deposition, and many mature fibroblasts.^39^

 In conclusion, the sponge-scaffold system must resemble the treated tissue’s physiological condition, including the consistency and pH to support cell proliferation and tissue repair. Besides, the sponge scaffold system must have the ability to absorb water. This ability shows that the scaffold can absorb and retain oxygen, nutrients, and other important factors from the surrounding fluids to support tissue regeneration.^107-109^ A scaffold must survive as a medium for cell colonization, proliferation, and differentiation during therapy and be completely degraded when the regeneration process ends. The internal content, polarity, and ability to absorb water all influence scaffold degradation.^107^

###  Devices

 Apart from using the excipient as a base, several devices are generally used to assist the administration of supplies to the target network. These devices have several advantages, including direct delivery to the target tissue, the ability to adjust the dose and maintain sterility. However, to be delivered, each device necessitates specific characteristics of the preparation. In situ gel or viscoelastic systems are required when using devices requiring the preparation to pass through a small space, such as a nozzle on a spray device or a needle on a syringe.^52,88,110,111^ Likewise, nebulizers and inhalers require the preparation to be in liquid or dry powder form.^112-114^ The following are some of the commonly or potentially used devices in delivering secretome-containing preparations.

#### Eye drops

 Secretome therapy in damaged eye tissue has been extensively studied and shows promising potential.^37,115–117^ One of the most common forms of delivery for ocular treatment is eye drops. The eye drop is the most convenient route and improves patient compliance compared to other conventional topical preparations. The eye drop system has been evolved over the last decade and can now contain in situ solutions of films, nanoparticles, or a combination of both.^118–120^ Some polymers, such as alginate,^121,122^ collagen,^123,124^ chitosan,^125-127^ cellulose derivatives,^128-130^ cyclodextrin^131,132^, gellan gum,^133-135^ pectin,^136^ poloxamer,^137-139^ and polyacrylic acid,^114,140^ have in situ gel/film and biocompatible properties, as well as the ability to be used as a biomedicine base. Some have even been successfully formulated into nanoparticles in situ gel drug delivery systems for ocular therapy.

#### Nebulizer/Inhaler

 Several studies on the use of secretomes by inhalation have demonstrated good respiratory organ repair, even in diseases previously only handled by transplantation, such as idiopathic pulmonary fibrosis.^32^ Secretome inhalation has also been proposed as a treatment for COVID-19 patients suffering from acute respiratory distress syndrome and is said to replace a ventilator’s role.^141^ Secretome can be delivered using a nebulizer or inhaler in the form of a solution or dry powder.^112-114^ The dry form of the secretome is more stable, scalable, and still provides excellent therapeutic efficacy.^142-145^

#### Topical sprays

 Topical sprays are better than conventional topical preparations because of their easier use, low incidence of irritation, sterility of the preparation, excellent coverage of the skin or wound, even distribution of drugs, and adjustable dosage. In situ film or viscoelastic dosage forms can be conveyed using a topical spray system. Film-forming topical spray can increase drug retention and higher drug penetration due to the thin film formed compared to using patches.^146^

#### Syringe

 Injectable hydrogel is the most widely used method in delivering secretome because it delivers the preparation directly to the internal tissues. This system also ensures that the therapeutic dosage is administered. However, if the secretome is not prepared in an adhesive matrix system, its bioavailability will still be below. This system is also limited in giving repeated doses, necessitating a matrix system capable of controlling secretome release.^147^

#### TheraCyte

 TheraCyte is a cell encapsulator device that has an outer neovascularization-promoting membrane and an inner immunoprotective membrane. TheraCyte has been used widely as a cell delivery system for various therapeutic purposes. TheraCyte is effective in maintaining the quality of insulin-producing cells during implantation. In the treatment process, this therapy does not cause or stimulate unwanted cells or tumors. The encapsulated cells will also not leak out of the TheraCyte membrane, and only the secretome or cell’s product will be released.^148-150^ The tissue form of syngeneic and allogeneic ovarian therapy was successfully implanted using this device. It reduced serum FSH levels in ovariectomized mice from 60-70 ng/mL to 30-40 ng/mL after 30 days of administration.^151^

 Kompa et al discovered that using TheraCyte as a cell protector for human cardiac stem cells resulted in cell viability and secretome release in a sustained pattern after four weeks of implantation. Some secretome contents have been found in the heart after four weeks of implantation, including those involved in inflammation, immunoregulation, cell survival, angiogenesis, tissue remodeling, and fibrosis, which can stimulate heart repair after myocardial infarction.^61^ Compared to repeated doses, a slowed release system will increase patient comfort during the treatment.

###  Secretome product

 Only a few companies have declared their products containing secretomes for regenerative therapy, including Regeneus and Pharmaexceed. Regeneus has a product containing a secretome called Sygenus. This product is a cell-free serum or gel used to treat pain, inflammation, and subsequent tissue repair. The secretome of Sygenus is derived from adipose MSCs. Sygenus itself is ready for phase one clinical testing.^152^

 Pharmaexceed itself is a biotechnology company that develops secretome or exosome-based products for therapeutic applications. The product name is Lyosecretome. This product is indicated to treat various acute, chronic, and degenerative pathologies of inflammatory and immune nature, musculoskeletal, cardiovascular, neurological, digestive, tegumentary, respiratory, and genitourinary. It is said that Lyosecretome can be combined with fibroin silk and alginate as a biotherapeutic product.^153^

###  Author perspective

 Previous studies have focused mostly on optimizing secretome production, control of release, and effectiveness of therapy. Secretome stability testing is rarely done or reported. A good delivery system must be capable of delivering the secretome and maintaining its stability for an extended period during storage. Therefore, product shelf-life, product stability, and quality control studies must be carried out in further research. Several previously described polymers can be studied further to answer how the system can maintain the secretome’s stability. One of the natural polymers that can also be used to maintain secretome quality is chitosan. Chitosan has antimicrobial, antioxidant, and anti-tumor properties, and it is biocompatible with cells. Chitosan naturally also has in situ film/gel properties to easily convey using injection, drop, and topical spray devices.

 Although some of the biomolecules have been encased in exosomes, some of the secretome’s free protein content can be degraded by enzymes when given directly to the body, necessitating in vivo stabilization. One method that can be done is adding an antiproteolytic agent, but this method can affect the systemic metabolic system.^154^ Another solution is to use a liposome. The liposome can protect the cargo from chemical, immune, and enzymatic reactions. This is due to the enzyme’s inability to recognize the liposome-coated protein. Liposomes can also transport drugs across the blood-brain barrier to the central nervous system.

 One of the challenges is oral delivery of the secretome. Some diseases such as wounds and gastric and intestinal cancer will be better treated with secretome. No studies have yet been conducted to design secretome delivery systems for oral therapy. In addition, most of the literature designs delivery systems that are directly inserted into the systemic/organ, so it is difficult to find literature studying the penetration of the secretome from one tissue to another with the aid of delivery systems.

 In clinical trials, some of the patients sometimes give different responses. This may be due to differences in the culture medium conditions and the secretome source, apart from the carrier. For that reason, in the future, it will be necessary to standardize secretome content based on the source.

## Conclusion

 Secretome requires a localized delivery system with continuous release to improve its effectiveness. Secretome delivery for various organ therapies necessitates the use of different delivery systems and bases. Coating, muco-, and cell-adhesive systems are required for systemic delivery and to prevent metabolism. The lyophilized form is required for inhalational delivery, and the lipophilic system can deliver secretomes across the blood-brain barrier. Nano-sized encapsulation and surface-modified systems can deliver secretome to the liver and kidney.

 Several dosage forms can be used, such as fibrous, in situ, or viscoelastic hydrogel, sponge-scaffold, beads powder/suspension, and bio-mimetic coating fabricated from natural, semi-synthetic, or synthetic polymers. These dosage forms can be administered using devices such as a sprayer, eye drop, inhaler, syringe, and implant to improve their efficacy through dosing, direct delivery to target tissues, preserving stability and sterility, and reducing the immune response.

## Acknowledgments

 AKU thanks the Chulalongkorn University’s Graduate Scholarship Programme (ASEAN or Non-ASEAN scholarship) for funding his Ph.D study in Thailand.

## Competing Interests

 The author declares that there is no conflict of interest. The scholarship funder had no role in the design of the study; in the collection, analyses, or interpretation of data; in the writing of the manuscript; or in the decision to publish the results.

## Ethical Approval

 There is no animal experiment carried out for this article.
